# A Rare Case of Terminal Ileal Diverticulitis: Clinical Presentation, Diagnosis, and Management

**DOI:** 10.7759/cureus.81607

**Published:** 2025-04-02

**Authors:** Danielle A Cabiran, Sarina Nikzad, Michael J Padron, Ishaan Dutta, Ryan Lusk

**Affiliations:** 1 Internal Medicine, Liberty University College of Osteopathic Medicine, Lynchburg, USA; 2 Medicine, Liberty University College of Osteopathic Medicine, Lynchburg, USA; 3 Obstetrics and Gynecology, Liberty University College of Osteopathic Medicine, Lynchburg, USA; 4 Internal Medicine, Sentara Obici Hospital, Suffolk, USA

**Keywords:** abdominal sepsis, gastro-intestinal, internal med, manage, small bowel diverticulitis

## Abstract

Diverticulitis is an acute inflammatory condition that typically affects the colon. Diverticulitis of the ileum is rare, with most cases reported in the terminal ileum, and is often triggered by an underlying infection, obstruction, or ischemia. Clinically, ileal diverticulitis presents with symptoms similar to colonic diverticulitis, such as abdominal pain, fever, and constipation. Symptoms can also mimic other conditions, including Crohn's disease and appendicitis. Thus, diagnosis is difficult and often delayed, leading to abscess, fistula formation, and acute abdomen. Diagnosing ileal diverticulitis requires a high clinical suspicion, which is depicted in this case of terminal ileal diverticulitis complicated by sepsis and the patient’s underlying medical comorbidities.

## Introduction

Diverticulitis is an inflammation of diverticula - small pouches that can form in the walls of the gastrointestinal (GI) tract. Most diverticula are found in the colon, particularly the sigmoid colon; however, they have also been identified in the small bowel. Small intestinal diverticula are commonly found in the duodenum [1]. The presence of diverticula in the terminal ileum is exceedingly rare and often asymptomatic [2]. However, they can lead to complications, including small intestinal bacterial overgrowth, lower GI bleeding, small bowel obstruction, and acute diverticulitis [1,3]. Diagnosing acute small bowel diverticulitis can be challenging, as it presents with nonspecific symptoms such as bloating, intermittent abdominal pain, constipation, and diarrhea [2]. Additionally, the symptoms may overlap with other GI conditions, such as Crohn’s disease, appendicitis, and small bowel obstruction [4]. Therefore, while the clinical presentation may raise suspicion, the diagnosis of small bowel diverticulitis is ultimately confirmed through imaging [5]. Computed tomography (CT) is the preferred imaging modality for diagnosing small bowel diverticulitis, typically demonstrating a thickened ileal wall, surrounding mesenteric fat stranding, and possible abscess formation. Sonography may also be used, though its role is more limited, primarily detecting bowel wall thickening and localized inflammation in experienced hands [2].

Accurate diagnosis of terminal ileum diverticulitis is crucial for appropriate treatment, as early surgical intervention may be required for complications such as perforation and obstruction. For uncomplicated cases, conservative management is found to be effective, which underscores the importance of obtaining an accurate diagnosis of acute GI conditions before initiating treatment [6].

Herein, we present a 77-year-old male patient undergoing chemotherapy for multiple myeloma, with a history of chronic congestive heart failure, type II diabetes mellitus, hypertension, and prostate cancer in remission, who presented with sepsis secondary to acute diverticulitis of the terminal ileum, which was successfully managed with medical intervention. This rare presentation, further complicated by the patient’s comorbidities, highlights the need for maintaining a high clinical suspicion in patients with nonspecific GI presentations.

## Case presentation

A 77-year-old man with a history of congestive heart failure, type II diabetes mellitus, hypertension, prostate cancer in remission, and multiple myeloma, for which he is undergoing chemotherapy, presented to the Emergency Department with fever, chills, and abdominal pain that began three days prior to arrival. The patient reported abdominal pain resembling constipation-associated discomfort, but with greater intensity. He had not had a bowel movement in three days. He denies any rectal bleeding, no bright red-colored stool, nor darkened, tarry stool. The patient is currently receiving chemotherapy; his schedule is 14 days on and 1 week off. The last cycle of chemotherapy was five days before hospital admission. 

Physical examination revealed a body temperature of 101.7°F, heart rate of 70 bpm, respiratory rate of 22 breaths per minute, and blood pressure of 110/55 mmHg. Blood culture was positive for gram-positive cocci. Abdominal tenderness to the bilateral lower quadrants was noted upon deep and light palpation. Associated symptoms were positive for chills, light-headedness, and shortness of breath.

Tables 1-2 show the critical lab values for the patient. Based on the Systemic Inflammatory Response Syndrome (SIRS) criteria, the patient demonstrated physical exam findings that showed signs of sepsis.

**Table 1:** Complete blood count

Complete Blood Count	Lab Value	Reference Range
Hemoglobin	8.9 g/dL	13.5-17.5 g/dL
Red blood cell count	3.25 M/uL	4.3-5.9 M/uL
Hematocrit	29.5%	41-53%
White blood cell	5.8 K/uL	4.5-11 K/uL
Segmented neutrophils	98%	54-62%
Lymphocytes	1 K/uL	0.8-4.8 K/uL

**Table 2:** Cardiac biomarkers

Cardiac Biomarkers	Lab Value	Reference Range
Troponin	29 ng/mL	<0.04 ng/mL
Lactate	3.3 mmol/L	0.5-2.2 mmol/L

**Figure 1:**
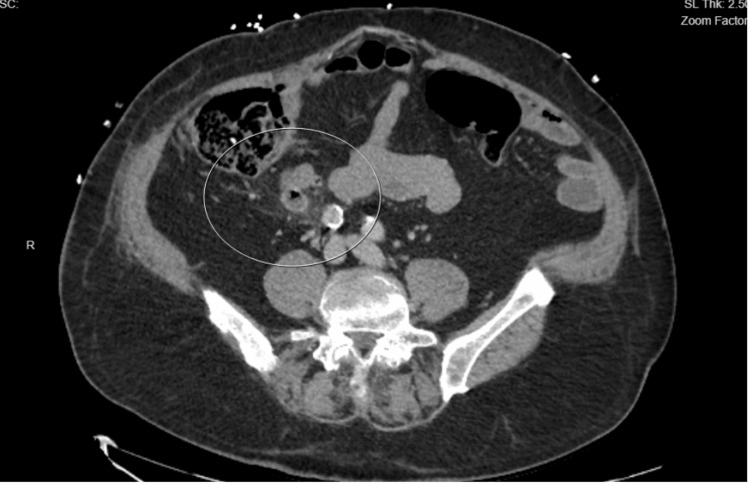
CT scan of diverticulitis of terminal ileum CT, Computed Tomography

Abdominal and chest CT was performed, which showed small diverticula protruding from the terminal ileum, with surrounding low-grade inflammatory changes consistent with diverticulitis of the terminal ileum (Figure 1).

The patient was started on intravenous (IV) fluids and IV piperacillin/tazobactam (Zosyn), switched to a clear liquid diet, and treated for pain control. Zosyn was the broad-spectrum antibiotic of choice due to its effect on gram-negative rods and anaerobic bacteria. After two days, he was advanced to solid food, while IV Zosyn was continued. After three days, he was discharged on oral ciprofloxacin 500 mg and metronidazole 500 mg, with a follow-up with GI. 

## Discussion

Ileal diverticula are caused by herniation of mucosa and submucosa through the intestinal wall, secondary to focal increases in intraluminal pressure and muscular wall weakness [7]. Due to limitations of endoscopic access and conventional imaging techniques, ileal diverticula are often identified intraoperatively [8,9]. Non-invasive modalities, such as video capsule endoscopy, have been used to identify Meckel’s diverticulum, but there have been no reports on their use for diagnosing ileal diverticula [10].

The symptoms of ileal diverticula are non-specific and include bloating, intermittent abdominal pain, constipation, and diarrhea; however, most cases are asymptomatic. This picture overlaps with other GI conditions and can delay reaching the diagnosis. Reported complications include acute diverticulitis, bleeding, and intestinal obstruction [9]. Ileal diverticulitis is the main complication reported in clinical studies, and its presentation can overlap significantly with other GI conditions, such as Crohn’s disease, appendicitis, and small bowel obstruction.

The most common presenting symptom is right lower quadrant pain, as seen in our patient [2]. This nonspecific presentation can delay diagnosis, which carries the risk of serious and life-threatening complications, such as intestinal perforation, acute abdomen, abscess formation, and fistulae [7,8]. Our patient's fever and three-day abdominal pain raised suspicion for sepsis secondary to a GI process, which was further investigated with abdominal CT.

Studies have emphasized the use of imaging for early diagnosis of this rare entity. The patient’s CT demonstrated mild inflammatory changes surrounding a small diverticulum in the terminal ileum, which supports diverticulitis as the source of sepsis. CT findings of acute diverticulitis typically show colonic wall thickening, pericolonic fat stranding, and outpouching in the colonic wall where the diverticulum is, all of which were seen in our patient's CT [11].

While imaging did not reveal frank perforation, the low-grade inflammatory changes surrounding the diverticulum suggest that bacterial translocation or microperforation may have occurred. This, combined with the patient's immunocompromised state, likely precipitated a SIRS, as evidenced by his presentation and blood culture result.

Similar to colonic diverticulitis, diverticulitis of the terminal ileum can be managed medically or surgically, depending on the patient's presentation. Many studies endorse conservative management of uncomplicated presentations, while reserving surgical intervention for complicated cases, such as those presenting with frank perforation, hemorrhage, or obstruction [2,3,5,6]. Conservative intervention consists of bowel rest, hydration, and antibiotics on a case-by-case basis, which can be done in an outpatient setting [6]. Despite the absence of frank perforation or abscess formation, the presence of inflammatory changes surrounding the diverticulum suggests bacterial translocation or microperforation, leading to sepsis. Given his immunocompromised status and systemic inflammatory response, hospitalization was warranted for close monitoring, along with IV fluids, IV antibiotics, and pain control.

## Conclusions

This case highlights the rare occurrence of terminal ileal diverticulitis complicated by sepsis in a patient with multiple, significant comorbidities. Prompt diagnosis, using imaging, combined with conservative medical management, resulted in successful resolution of the condition. This case underscores the importance of maintaining a high index of suspicion for ileal diverticulitis in patients presenting with nonspecific abdominal symptoms. Early recognition and appropriate treatment are critical to preventing severe complications and ensuring favorable patient outcomes.
